# Analysis of the transcriptional responses in inflorescence buds of *Jatropha curcas* exposed to cytokinin treatment

**DOI:** 10.1186/s12870-014-0318-z

**Published:** 2014-11-30

**Authors:** Mao-Sheng Chen, Bang-Zhen Pan, Gui-Juan Wang, Jun Ni, Longjian Niu, Zeng-Fu Xu

**Affiliations:** Key Laboratory of Tropical Plant Resources and Sustainable Use, Xishuangbanna Tropical Botanical Garden, Chinese Academy of Sciences, Menglun, Yunnan 666303 China; University of Chinese Academy of Sciences, Beijing, 100049 China; School of Life Sciences, University of Science and Technology of China, Hefei, Anhui 230027 China

**Keywords:** Cytokinin, Flowering, Physic nut, Phytohormone, Woody plant, Microarray

## Abstract

**Background:**

*Jatropha curcas* L. is a potential biofuel plant. Application of exogenous cytokinin (6-benzyladenine, BA) on its inflorescence buds can significantly increase the number of female flowers, thereby improving seed yield. To investigate which genes and signal pathways are involved in the response to cytokinin in *J. curcas* inflorescence buds, we monitored transcriptional activity in inflorescences at 0, 3, 12, 24, and 48 h after BA treatment using a microarray.

**Results:**

We detected 5,555 differentially expressed transcripts over the course of the experiment, which could be grouped into 12 distinct temporal expression patterns. We also identified 31 and 131 transcripts in *J. curcas* whose homologs in model plants function in flowering and phytohormonal signaling pathways, respectively. According to the transcriptional analysis of genes involved in flower development, we hypothesized that BA treatment delays floral organ formation by inhibiting the transcription of the A, B and E classes of floral organ-identity genes, which would allow more time to generate more floral primordia in inflorescence meristems, thereby enhancing inflorescence branching and significantly increasing flower number per inflorescence. BA treatment might also play an important role in maintaining the flowering signals by activating the transcription of *GIGANTEA* (*GI*) and inactivating the transcription of *CONSTITUTIVE PHOTOMORPHOGENIC 1* (*COP1*) and *TERMINAL FLOWER 1b (TFL1b*). In addition, exogenous cytokinin treatment could regulate the expression of genes involved in the metabolism and signaling of other phytohormones, indicating that cytokinin and other phytohormones jointly regulate flower development in *J. curcas* inflorescence buds.

**Conclusions:**

Our study provides a framework to better understand the molecular mechanisms underlying changes in flowering traits in response to cytokinin treatment in *J. curcas* inflorescence buds. The results provide valuable information related to the mechanisms of cross-talk among multiple phytohormone signaling pathways in woody plants.

**Electronic supplementary material:**

The online version of this article (doi:10.1186/s12870-014-0318-z) contains supplementary material, which is available to authorized users.

## Background

*Jatropha curcas* L. (Euphorbiaceae) is a perennial bush or small tree that is widely cultivated in tropical and subtropical climates. The oil content of *J. curcas* seeds is 30–40%, and *J. curcas* grows well on marginal lands, avoiding competition with food production. Thus, *J. curcas* is a potential biofuel plant [1,2]. However, its potential as a biofuel plant is limited by its poor seed yield [3]. Research into the biological and genetic factors that contribute to seed production in *J. curcas* is necessary for genetic improvement by conventional and molecular breeding approaches [4-6]. *J. curcas* is a monoecious plant with unisexual flowers: both male and female flowers are borne on the same racemose inflorescence. Each inflorescence has approximately 15 female flowers and 13 pieces of fruit under normal growth conditions [7]. Therefore, to improve the seed yield of *J. curcas*, generating sufficient female flowers is crucial. A previous study [7] applied exogenous cytokinin (6-benzyladenine, BA) to *J. curcas* inflorescence buds and obtained a 9.4-fold increase in the number of female flowers per inflorescence and a 2.3-fold increase in seed yield, providing a promising strategy for improving the seed yield of *J. curcas*.

Cytokinins are an important class of phytohormones that were first discovered to promote cell division in tobacco tissues in 1955 [8]. Cytokinins are involved in many important aspects of plant growth and development, e.g. promoting vascular cambium activity, controlling organ development, and regulating shoot and root branching, as well as responding to biotic and abiotic stresses [9-11]. They also play important roles in maintaining the activity and function of the shoot apical meristem (SAM) [9,12]. The SAM comprises a small population of dividing cells located at the shoot tip, and is responsible for the initiation of all the aerial parts of plants, including the reproductive organs [13]. At least three possible pathways have been identified for maintaining the homeostasis of stem cells, which is necessary for meristem activity in *Arabidopsis* [14]. A number of genes, such as *SHOOT MERISTEMLESS* (*STM*), *WUSCHEL* (*WUS*), *CLAVATA* (*CLV*), *LONELY GUY* (*LOG*), *AINTEGUMENTA* (*ANT*), *ANT-like 6* (*AIL6*), *ANT-like 7* (*AIL7*), are involved in this process [14-22].

*KNOTTED1-like homeobox* (*KNOXI*) increases cytokinin biosynthesis by promoting the expression of *ISOPENTENYL TRANSFERASE* (*IPT*), which encodes a rate-limiting enzyme in cytokinin biosynthesis. The application of exogenous cytokinin or the expression of a cytokinin biosynthesis gene rescued a *stm* mutant [22,23]. WUS directly represses the expression of *ARABIDOPSIS RESPONSE REGULATOR 5, 6, 7,* and *15 (ARR5, 6, 7, and 15)*, which are negative regulators in the cytokinin signaling pathway, and cytokinin signaling activated the expression of *WUS* through both CLV-dependent and CLV-independent pathways [24-26]. LOG catalyzes the final step of cytokinin biosynthesis within the rice meristem, and *log* mutants have defects in shoot meristem function, showing small panicles, and abnormal floral organs and branching patterns [22]. Cytokinin oxidase/dehydrogenase (CKX) catalyzes the degradation of cytokinin to regulate the activity of reproductive meristems in *Arabidopsis*. A *ckx3ckx5* double mutant produced larger inflorescences and floral meristems, and had an approximately 55% higher seed yield [27,28]. In rice, a mutant with a reduced expression of *OsCKX2* accumulated cytokinin in inflorescence meristems, which contributed to an increased number of spikelets and reproductive organs [29]. Therefore, endogenous cytokinins are assumed to promote the size of reproductive meristems and the number of reproductive organs by initiating more floral primordia in the SAM [28]. In addition, cytokinins influence the switch from the vegetative to the reproductive phase and are involved in the regulation of floral development [30-34]. The application of cytokinin promoted *Arabidopsis* flowering by activating *TWIN SISTER OF FT* (*TSF*), *FD*, and *SUPPRESSOR OF OVEREXPRESSION OF CONSTANS 1* (*SOC1*). By contrast, *FLOWERING LOCUS T* (*FT*) was not required, suggesting that *FT* and *TSF* belong to distinct floral signal pathways that respond to different environmental and internal signals [11].

Our previous study showed that exogenous application of BA significantly increased the total number of flowers and the proportion of female flowers in *J. curcas* [7]. To understand the molecular mechanism of cytokinin action in *J. curcas* inflorescence buds, we analyzed the dynamic changes in gene expression at different time points after BA treatment using a microarray. Differentially expressed genes involved in the metabolism and signaling of cytokinin and other phytohormones, flowering and floral organ development, and cell division were identified. Our results provide a basis for determining the mechanism of cytokinin action on floral development in *J. curcas*.

## Results and discussion

### Effects of 6-benzyladenine (BA) on flowering and fruiting in *J. curcas*

**Figure 1:**
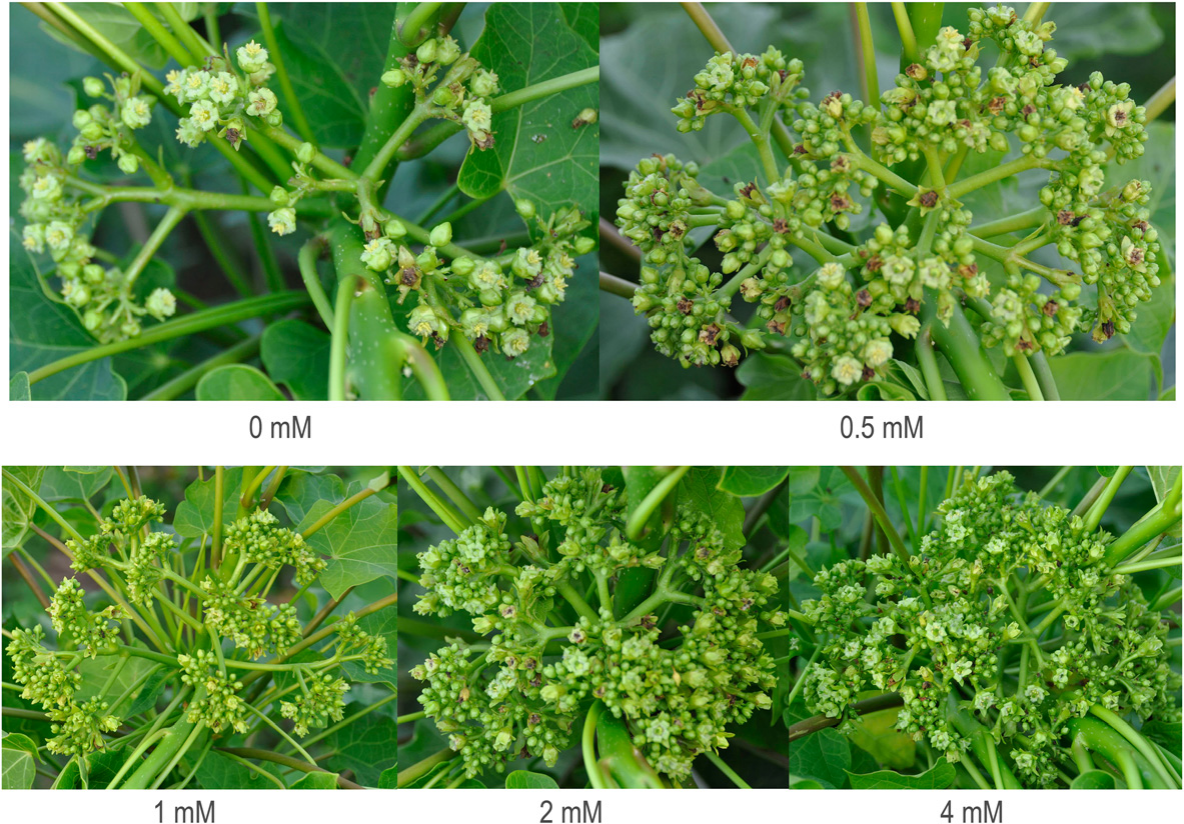
**Effects of 6-benzyladenine (BA) on flower development in** ***J. curcas.*** Each *J. curcas* inflorescence bud was treated with a solution of BA (0, 0.5, 1.0, 2.0, or 4.0 mM). Each group included 30 inflorescence buds.

**Figure 2:**
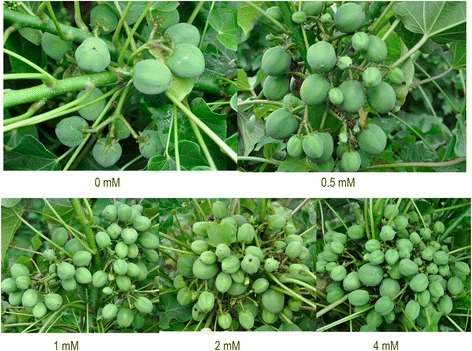
**Effects of 6-benzyladenine (BA) on fruit development in** ***J. curcas.*** Each *J. curcas* inflorescence bud was treated with a solution of BA (0, 0.5, 1.0, 2.0, or 4.0 mM). Each group included 30 inflorescence buds.

**Figure 3:**
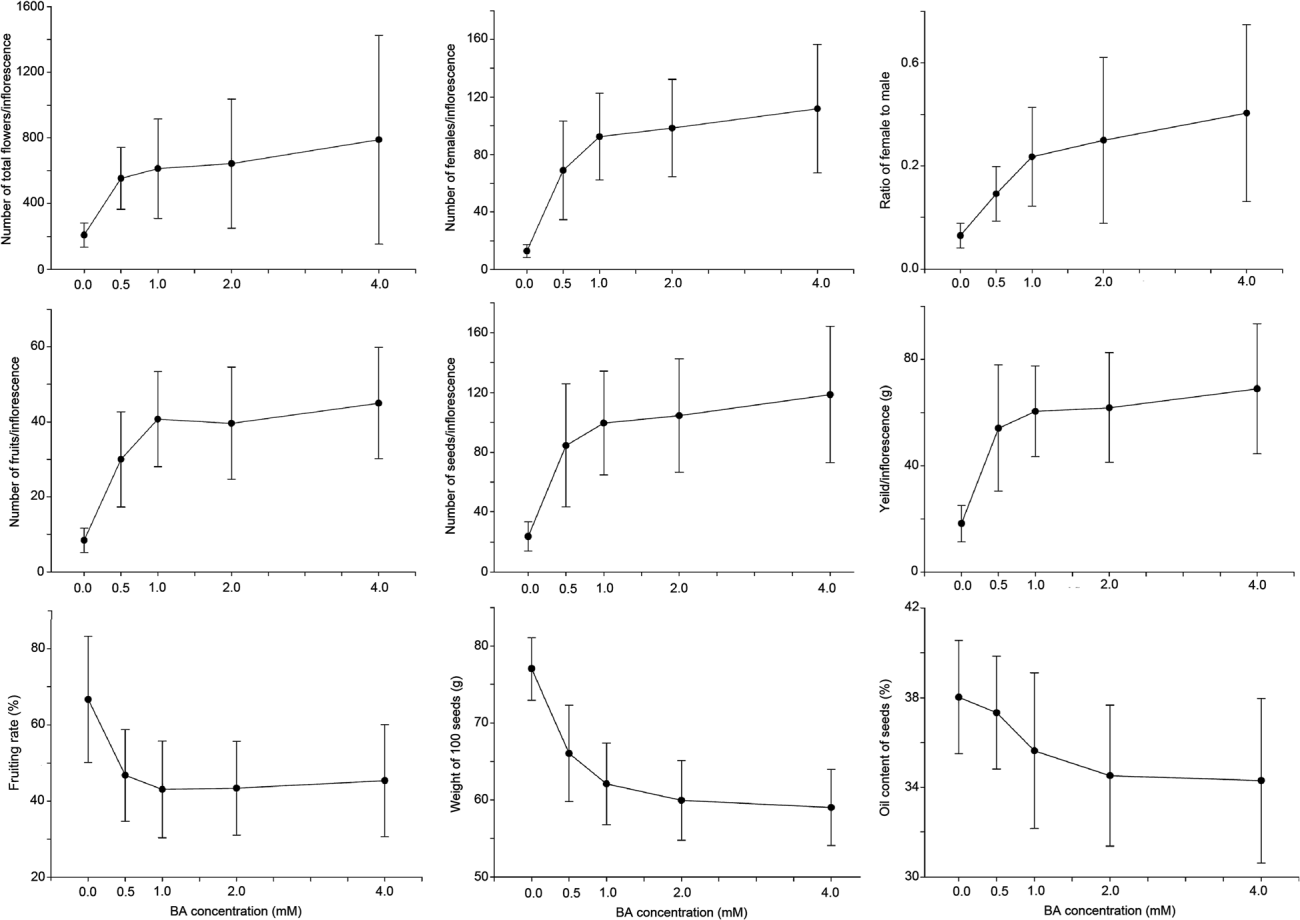
**Effects of 6-benzyladenine (BA) on flowering and fruiting in** ***J. curcas.*** Values are provided as means ± standard deviations (n = 30).

The application of BA changed the flowering characteristics of *J. curcas* and remarkably increased the number of female flowers to improve seed yield [7]. To select a suitable concentration of BA for this study, we treated *J. curcas* inflorescence buds with 0, 0.5, 1.0, 2.0, or 4.0 mM of BA, and then surveyed the total flower number, female flower number, ratio of female to male flowers, fruit number, fruiting rate, seed number, seed yield, weight of 100 seeds, and seed oil content per inflorescence. We confirmed that the application of BA is an effective way to significantly increase the number of female flowers and fruits, resulting in an increased seed yield in *J. curcas* (Figures 1 and 2). The total flower number, female flower number, ratio of female to male flowers, fruit number, seed number, and seed yield all increased with BA concentration from 0.5 to 4.0 mM, while the fruiting rate, weight of 100 seeds, and seed oil content decreased (Table 1). The numbers of female flowers, fruits, and seeds per inflorescence were 7.7-, 4.4-, and 4.0-fold higher, respectively, in the 4.0 mM BA treatment than in the control. As shown in Figure 3, 1.0 mM BA was a transition point in the biological response, and therefore was selected for use in subsequent experiments. Indeed, the effect of a single 1.0 mM BA treatment employed in this study was similar to that of three consecutive treatments at 1-day intervals with 160 mg/L (0.71 mM) BA [7]: both resulted in a 2.3-fold increase in final seed yield (Table 1).

**Table 1:** **Effects of 6-benzyladenine (BA) treatment on flowering and fruiting in** ***J. curcas***

**Concentration of BA (mM)**	**Number of flowers/inflorescence**	**Number of females/inflorescence**	**Ratio of female to male**	**Number of fruits/infructescence**	**Fruiting rate (%)**	**Number of seeds/infructescence**	**Weight of 100 seeds (g)**	**Yield/infructescence (g)**	**Oil content (%)**
0	207.1 ± 72.4	12.9 ± 4.5	0.1 ± 0.0	8.4 ± 3.2	66.7 ± 16.6	23.6 ± 9.6	77.0 ± 4.1	18.3 ± 6.8	38.0 ± 2.5
0.5	553.2 ± 189.0**	68.9 ± 34.4**	0.1 ± 0.1	30.0 ± 12.8**	46.7 ± 12.0**	84.5 ± 41.2**	66.1 ± 6.3	54.2 ± 23.8**	37.3 ± 2.5
1.0	612.7 ± 303.3**	92.3 ± 30.1**	0.2 ± 0.1*	40.7 ± 12.6**	43.0 ± 12.7**	99.6 ± 34.7**	62.1 ± 5.3	60.5 ± 17.0**	35.6 ± 3.5*
2.0	643.5 ± 394.4**	98.3 ± 33. 7**	0.3 ± 0.2*	39.6 ± 14.9**	43.4 ± 12.3**	104.6 ± 37.9**	59.9 ± 5.2**	61.9 ± 20.6**	34.5 ± 3.2**
4.0	789.1 ± 635.6**	111.8 ± 44.6**	0.3 ± 0.2**	45.0 ± 14.9**	45.4 ± 14.8**	118.6 ± 45.7**	59.0 ± 4.9**	69.0 ± 24.5**	34.3 ± 3.7**

Each treatment includes 30 inflorescences.

*indicates significantly different at *P* ≤0.05.

**indicates significantly different at *P* ≤0.01.

### Differentially expressed genes in response to the application of BA on inflorescence buds

**Figure 4:**
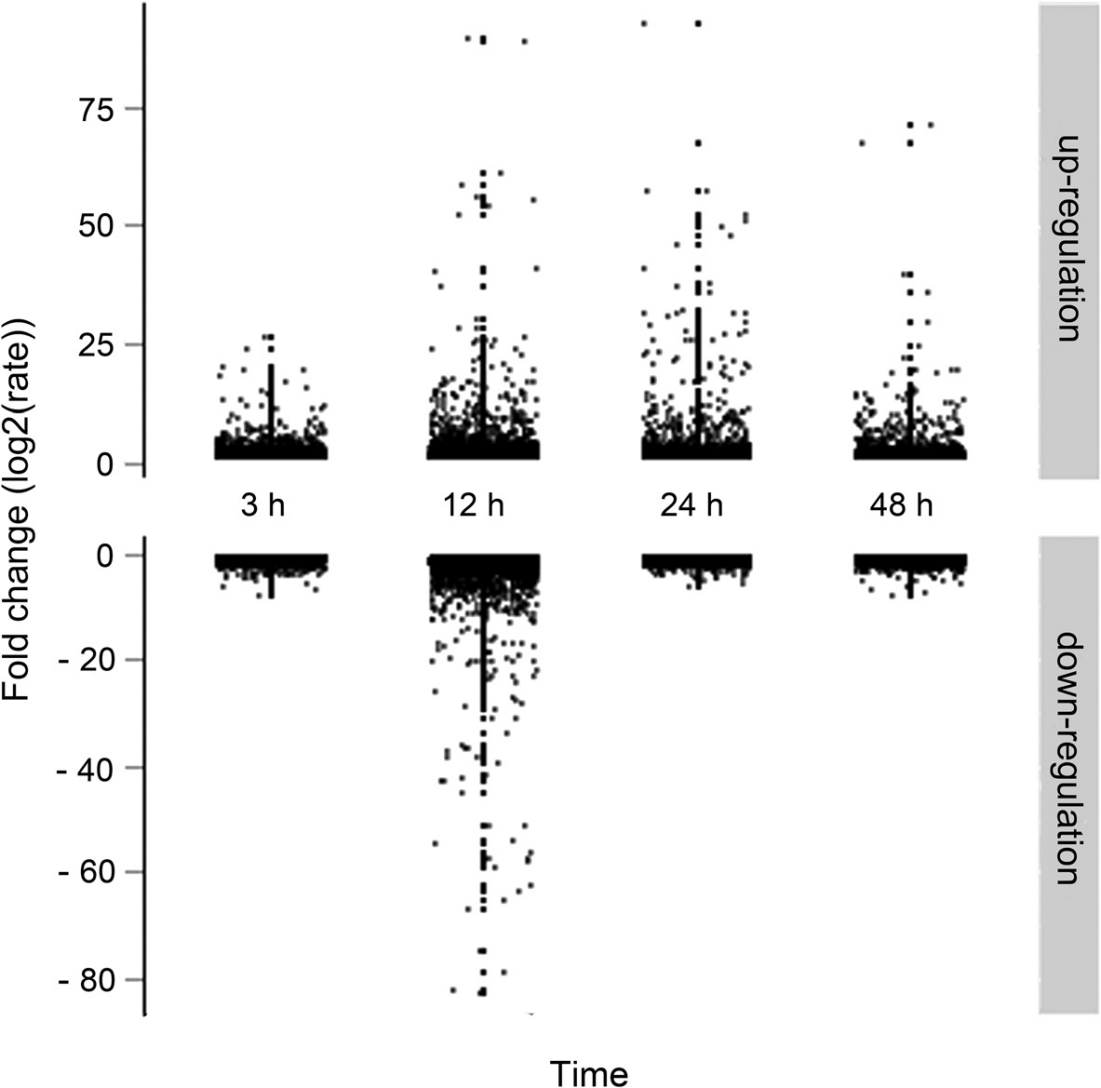
**Distribution of differentially expressed transcripts at 3-h, 12-h, 24-h, and 48-h time points after 6-benzyladenine (BA) treatment in** ***J. curcas.***

**Figure 5:**
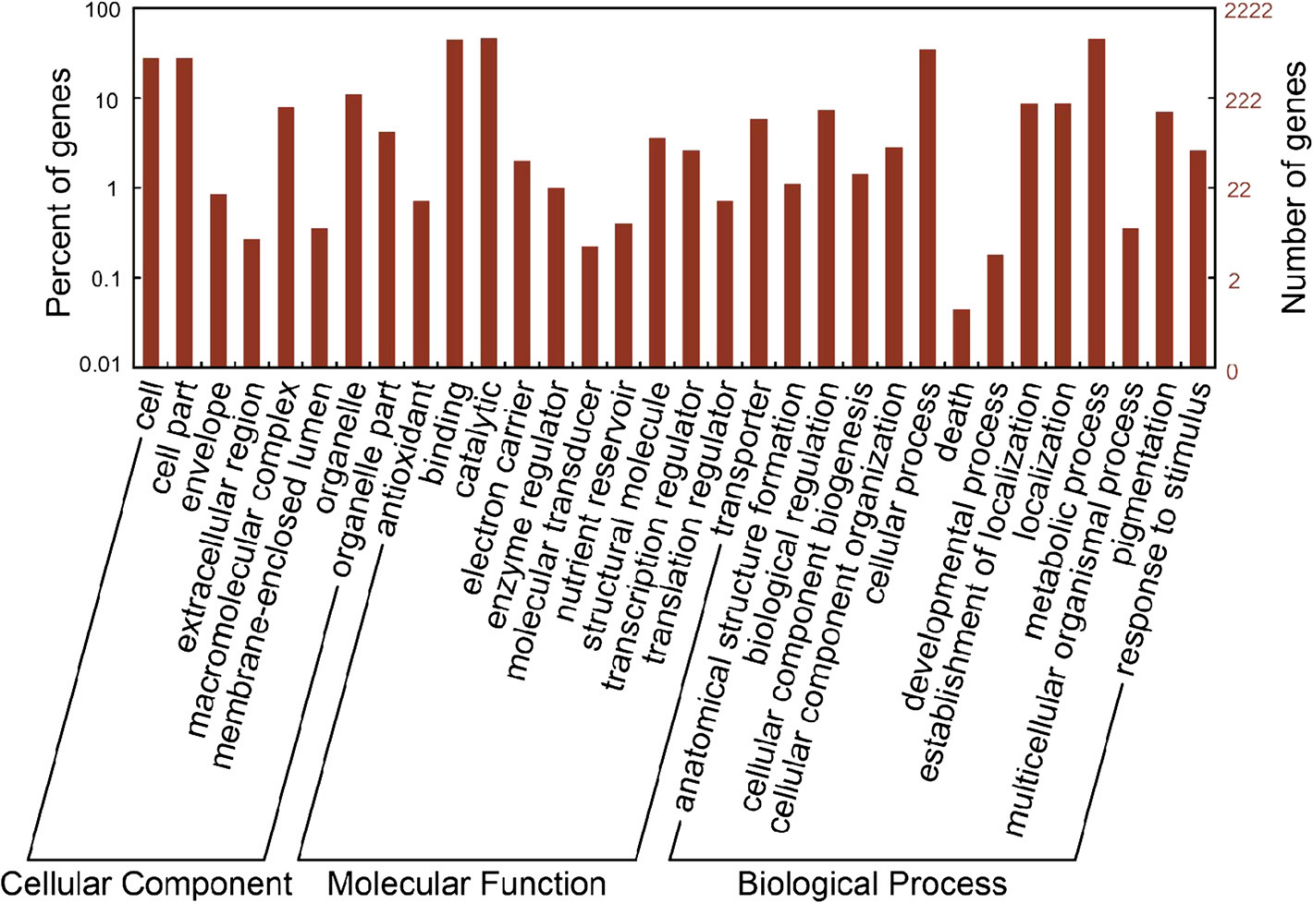
**Gene ontology categories of differentially expressed transcripts after 6-benzyladenine (BA) treatment in** ***J. curcas.*** 5,555 differentially expressed transcripts were annotated into 32 gene ontology categories in three main categories: biological process, cellular component, and molecular function.

To identify genes that responded to the application of BA, we designed 41,651 sequence-specific oligonucleotide probes representing 20,555 transcripts to monitor the transcription levels of genes in inflorescence buds at 0, 3, 12, 24, and 48 h after BA treatment using a microarray. 10,569 probes representing 5,555 transcripts changed significantly during the time course (Additional file 1). More transcripts were upregulated in inflorescence buds than were downregulated, except at the 12-h time point, when the greatest overall number of transcripts were differentially expressed among all the time points (Figure 4), indicating that this was an important phase in the response to exogenous cytokinin. Moreover, these differentially expressed transcripts (about 27%) were annotated into 32 gene ontology (GO) categories, as defined by various molecular functions and biological processes (Figure 5), indicating that determining the molecular mechanisms involved in the response to exogenous cytokinin in inflorescence buds will be difficult.

### Expression profiles of the differentially expressed genes in response to BA treatment

**Figure 6:**
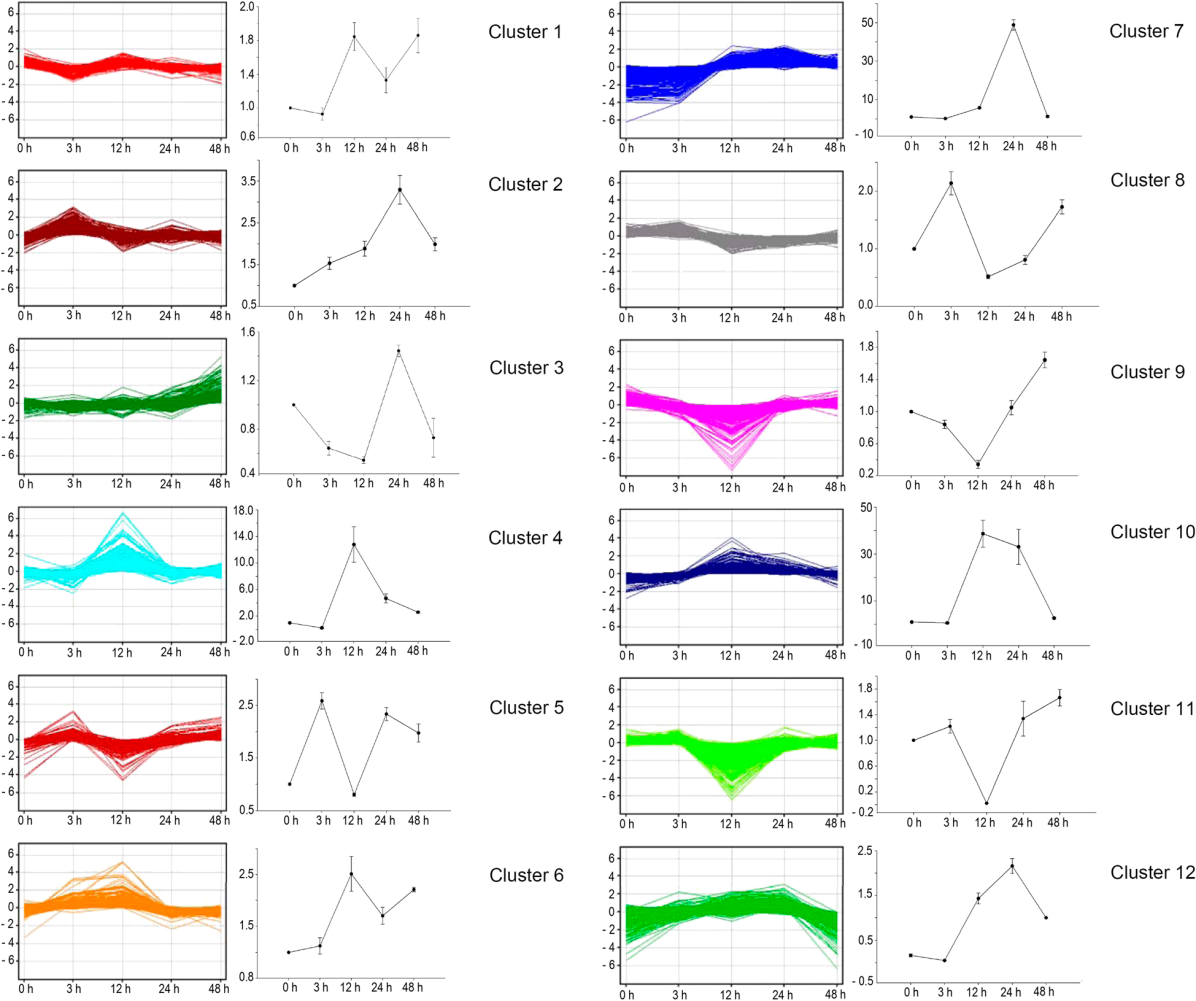
**Clustering analysis of differentially expressed transcripts with significant expression profile changes revealed by the microarray and qRT-PCR analysis.** All differentially expressed transcripts were clustered into 12 distinct temporal change patterns according to their expression profiles. The expressions of 12 selected transcripts representing the 12 expression patterns from the microarray analysis were confirmed by qRT-PCR.

To gain further insights into the genetic and biological processes involved in the response to BA application, the transcripts were clustered into 12 sets that represented distinct temporal expression patterns (Figure 6 and Additional file 2). Among these patterns, clusters 2 and 5 were upregulated and cluster 1 was downregulated at the 3-h time point, suggesting that these genes were induced early in the response to cytokinin; examples include calcium ion binding protein (CUST_18202), gibberellin 20-oxidase (CUST_5901), and gibberellin receptor GID1 (CUST_19936), respectively. The expressions of genes in clusters 4, 6, 9, 10 and 11 changed significantly from the 3-h to 24-h time points, indicating that significant transcriptional regulation occurred during this phase. Gene expression in clusters 7 and 8 changed distinctly from the 3-h to 12-h time points and remained constant until the 48-h time point. Moreover, the expression profiles of clusters 3 and 12 changed obviously between the 24-h and 48-h time points, suggesting that they might represent downstream genes in the BA response pathway.

To validate the results of the microarray analysis, we performed quantitative real-time reverse transcription PCR (qRT-PCR) analysis on 12 selected transcripts representative of the 12 clusters representing distinct expression patterns (Figure 6). The qRT-PCR expression profiles agreed with the profiles of their respective clusters; however, the sizes of the changes in expression at some time points were larger than in the clusters (Figure 6), suggesting that qRT-PCR was more sensitive than the microarray analysis. Based on the qRT-PCR results, we concluded that the expression profiles of transcripts in the clusters (Figure 6) accurately reflected temporal changes in the expressions of genes involved in the response to exogenous cytokinin.

### Functional analysis of the genes differentially expressed in response to BA application

**Figure 7:**
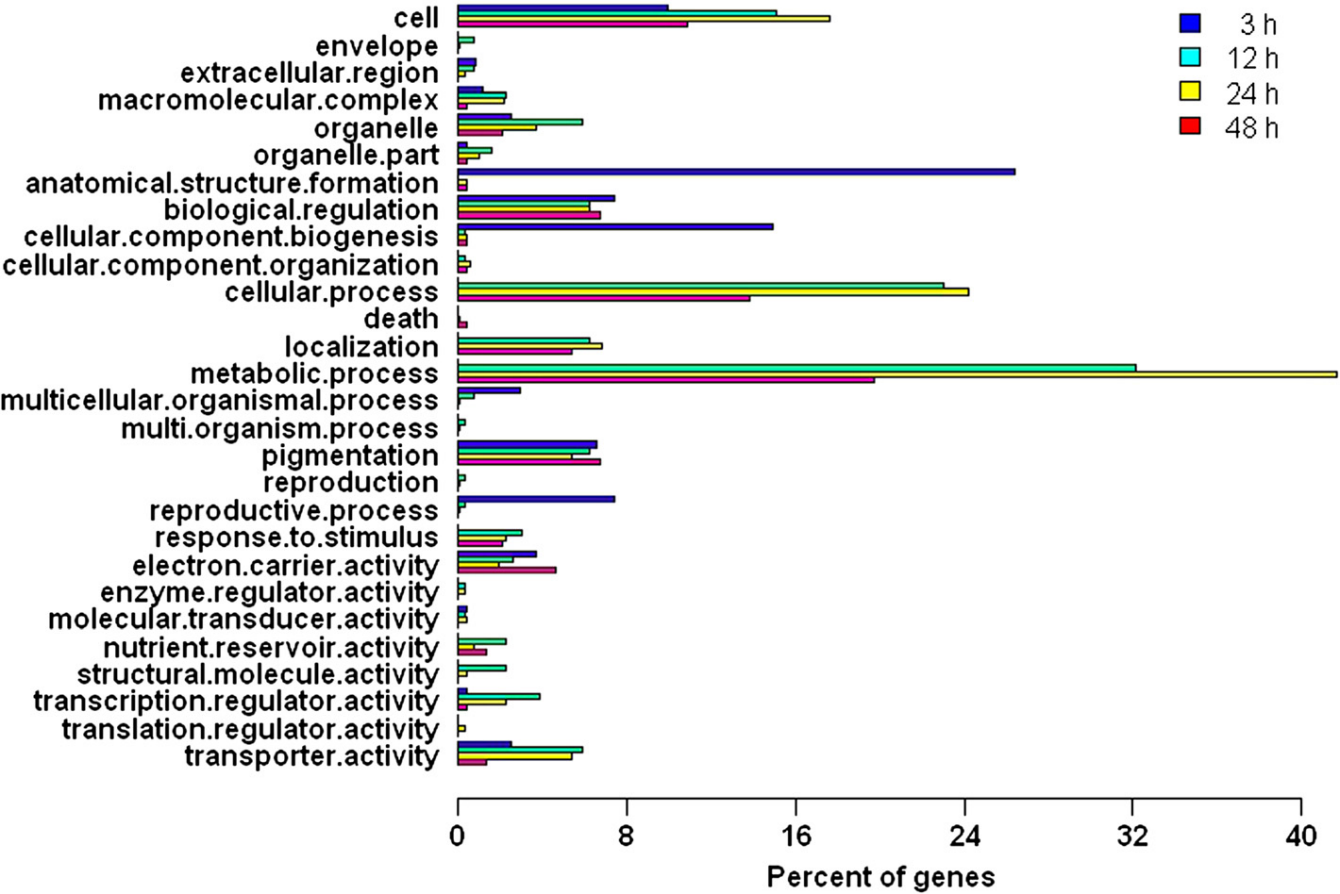
**Comparative analysis by gene ontology category of differentially expressed transcripts at different time points after 6-benzyladenine (BA) treatment in** ***J. curcas.*** The highest numbers of transcripts at the 3-h time point were in the “anatomical structure formation”, “cellular component biogenesis” and “reproductive process” categories. The numbers of transcripts were lowest in the “metabolic process”, “cellular process”, “localization” and “response to stimulus” categories. The numbers of transcripts were similar at four time points in the “cell”, “biological regulation”, “pigmentation”, “organelle”, “electron carrier activity” and “transporter activity” categories.

To understand the biological functions of the differentially expressed genes, those that changed ≥2-fold were categorized by GO analysis (Figure 7). Among all post-application time points, the numbers of transcripts in the “anatomical structure formation”, “cellular component biogenesis”, and “reproductive process” categories were highest at the 3-h time point (28%, 16% and 8%, respectively) and those in the “metabolic process”, “cellular process”, “localization” and “response to stimulus” categories were the lowest. However, the “cell”, “biological regulation”, “pigmentation”, “organelle”, “electron carrier activity” and “transporter activity” categories were similar at four time points. The results indicated that genes involved in the “anatomical structure formation”, “cellular component biogenesis”, and “reproductive process” functions were especially expressed at the 3-h time point to promote cell generation in the inflorescence, which might be the main factor driving the increase in flower number. We hypothesized that these specifically expressed genes were first induced by cytokinin, and then they in turn activated genes in the “metabolic process”, “cellular process”, “location” and “response to stimulus” categories to influence the growth and flowering of *J. curcas*.

### Transcriptional analysis of genes related to flower development

The application of BA had a significant effect on the flowering characters of *J. curcas*, which in turn resulted in an increased seed yield. Thirty-one transcripts in our dataset were homologous to genes related to flowering and flower development in *Arabidopsis* (Table 2). The expressions of nine genes were significantly differentially regulated (≥2-fold) by BA treatment. *CUP-SHAPED COTYLEDON 1* (*CUC1*) and *GIGANTEA* (*GI*) were upregulated, and *APETALA3* (*AP3*), *CONSTITUTIVE PHOTOMORPHOGENIC 1* (*COP1)*, *NGATHA 2* (*NGA2*), *SEPALLATA 1, 2,* and *3* (*SEP1, 2, and 3*), and *SEEDSTICK* (*STK*) were downregulated over the time course of the experiment.

**Table 2:** **Expression analysis of genes related to flowering in inflorescence buds of** ***J. curcas*** **after 6-benzyladenine (BA) treatment**

**Probe code**	**Gene name**	**The fold change of 3 h** ***vs*** **. 0 h**	**The fold change of 12 h** ***vs*** **. 0 h**	**The fold change of 24 h** ***vs*** **. 0 h**	**The fold change of 48 h** ***vs.*** **0 h**	**Gene ID**
CUST_14761	AGAMOUS	-1.21	-1.39	1.09	1.51	AT4G18960
CUST_225	AGAMOUS-LIKE 20	1.20	1.24	1.63	-1.35	AT2G45660
CUST_7819	APETALA 1	-1.43	-1.94	-1.28	-1.33	AT1G69120
CUST_10026	APETALA 2	-1.52	1.19	1.30	1.46	AT4G36920
CUST_10541	APETALA 3	-1.35	-4.33	-1.08	1.22	AT1G30950
CUST_34715	AUXIN RESPONSE FACTOR	-1.62	1.03	-1.34	1.66	AT5G60450
CUST_41624	AUXIN RESPONSE FACTOR 6	-1.07	-1.07	-1.03	-1.33	AT1G30330
CUST_31291	ASYMMETRIC LEAVES 1	-1.44	-1.13	-1.28	-1.20	AT2G37630
CUST_36781	CONSTITUTIVE PHOTOMORPHOGENIC 1	1.00	-2.20	-1.16	1.10	AT2G32950
CUST_11713	CRYPTOCHROME 1	-1.46	-1.97	-1.26	-1.25	AT4G08920
CUST_17122	CUP-SHAPED COTYLEDON 1	-1.44	2.37	2.59	2.72	AT3G15170
CUST_41631	CUP-SHAPED COTYLEDON 2	-1.29	-1.31	-1.31	-1.23	AT5G53950
CUST_7808	CUP-SHAPED COTYLEDON 3	-1.09	1.30	1.36	1.71	AT1G76420
CUST_15325	EARLY FLOWERING 8	-1.07	1.02	-1.07	1.06	AT2G06210
CUST_31664	ETTIN	-1.48	-1.12	-1.16	1.08	AT2G33860
CUST_41538	TOMATO MADS-box GENE 6	-1.26	-1.26	-1.13	-1.26	AT1G53160
CUST_31665	GIGANTEA	13.38	88.13	1.45	2.39	AT1G22770
CUST_39265	PISTILLATA	1.01	-1.01	1.29	1.34	AT5G20240
CUST_40397	NGATHA 2	-2.16	1.14	1.48	-1.19	AT3G61970
CUST_33212	PHYTOCHROME A	-1.22	-1.36	-1.15	-1.19	AT1G09570
CUST_14670	PIN-FORMED 1	1.20	1.40	1.24	1.15	AT1G73590
CUST_15904	SEPALLATA 1	-1.07	-2.72	-1.22	-1.22	AT4G34190
CUST_36715	SEPALLATA 2	-1.25	-2.42	-1.24	-1.06	AT3G02310
CUST_10402	SEPALLATA 3	-1.23	-2.98	-1.07	1.14	AT1G24260
CUST_5255	SOMATIC EMBRYOGENESIS RECEPTOR-LIKE KINASE 1	-1.09	1.15	-1.17	-1.18	AT1G71830
CUST_29859	SEUSS	-1.14	1.03	-1.05	-1.08	AT1G43850
CUST_32171	SPATULA	1.07	1.07	1.23	1.04	AT4G36930
CUST_19879	SEEDSTICK	-1.10	-3.30	1.00	1.55	AT4G09960
CUST_36747	SHOOT MERISTEMLESS	-1.07	-1.03	-1.03	-1.26	AT1G62360
CUST_1965	SHORT VEGETATIVE PHASE	-1.05	1.15	-1.08	-1.07	AT2G22540
CUST_32932	ULTRAPETALA 1	-1.67	1.08	-1.08	-1.10	AT4G28190
CUST_36773	TASSELSEED2	-1.21	-2.29	-3.44	-3.19	162460536

Interestingly, *GI*, a clock-associated protein that is involved in the control of circadian rhythms and regulating flowering time [35,36], was induced quickly by BA treatment (by the 3-h time point) and was expressed 88 times higher at the 12-h time point than at the 0-h time point. *CUC1*, belonging to the NAC family, which contributes to the formation of the SAM and the separation of cotyledons by activating *STM* in *Arabidopsis* [37,38], was induced between the 12-h and 48-h time points, indicating that *CUC1* helps to promote and maintain SAM formation to generate more floral primordia. However, *CUC2* and *CUC3* were insensitive to BA treatment in *J. curcas*. This is in contrast to observations in *Arabidopsis*, where *CUC2* and *CUC3*, but not *CUC1*, were upregulated by cytokinin in inflorescence meristems [39].

Among the floral organ-identity genes, *AP1*, an A-class gene in *Arabidopsis*, and *AP3* (B-class), *SEP1*, *SEP2*, and *SEP3* (E-class) [40] were downregulated at the 12-h time point, while *AP2* (A-class) and *AG* (C-class) [41] were insensitive to BA application. These results implied that BA treatment could suppress the expressions of A-, B- and E-class genes, which agreed with our observation that the flowering duration of inflorescences treated with BA was longer than that in the control (data not shown). Recently, *AP1* was observed to act upstream of cytokinin, regulating cytokinin levels by directly suppressing the cytokinin biosynthetic gene *LOG1* and activating the cytokinin degradation gene *CKX3* to suppress meristem activity in sepal axils [42].

**Figure 8:**
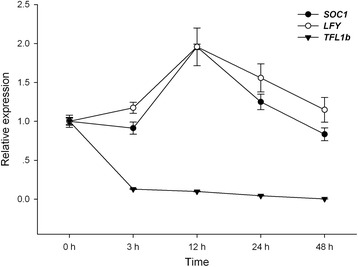
**Expression analysis of** ***LFY, SOC1*** **and** ***TFL1b*** **transcripts by qRT-PCR after 6-benzyladenine (BA) treatment in** ***J. curcas.*** *LFY* and *SOC1* were upregulated at the 12-h time point, and *TFL1b* was downregulated over the time course of the experiment. The *GHPDA* gene was used as an internal control.

We also verified the expressions of *SOC1*, *LEAFY* (*LFY*), and *TERMINAL FLOWER 1b (TFL1b*) in inflorescence buds of *J. curcas* after BA treatment by qRT-PCR. The transcript levels of *SOC1* and *LFY* were upregulated at the 12-h time point, and *TFL1b* was downregulated during the experiment (Figure 8). We hypothesized that BA treatment might contribute to the maintenance of the flowering signals by activating the expressions of *GI*, *LFY*, and *SOC1* and inactivating *COP1* and *TFL1b*. BA treatment may also delay the formation of floral organs by inhibiting the transcription of the A-, B- and E-class of floral organ-identity genes, which would allow more time to generate more floral primordia in inflorescence meristems, along with activating the expression of *CUC1*, which would result in a significant increase in flower number in *J. curcas*.

### Transcriptional analysis of genes involved in sex determination in *J. curcas*

As shown in Table 1, BA treatment significantly increased the ratio of female to male flowers, indicating that the cytokinin could affect the differentiation of male and female flowers in *J. curcas*. Among the oligonucleotide probes used in this study, CUST_36773 (Table 2) was homologous to the sex determination gene *TASSELSEED2* (*TS2*) of maize, which encodes a short-chain alcohol dehydrogenase that is required for carpel abortion in maize [43]. Upon BA treatment of inflorescence buds, the *J. curcas TS2* homolog (CUST_36773) was downregulated by 2.3-fold, 3.4-fold and 3.2-fold at the 12-h, 24-h and 48-h time points, respectively (Table 2). This result suggested that BA treatment activates the development of arrested pistil primordia in male flowers by repressing the expression of the *TS2* homolog in *J. curcas*, which resulted in an increase in the ratio of female to male flowers. Sex determination in plants, however, is a complex and dynamic process, and is influenced by genetic, hormonal and environmental conditions [44,45]. Further studies are required to elucidate the mechanism of sex determination in *J. curcas*.

### Transcriptional analysis of genes involved in cytokinin signaling in inflorescence buds

Endogenous cytokinins regulate many essential aspects of plant growth and development, and play a critical role in the formation and maintenance of the SAM. To further investigate the response mechanism of *J. curcas* inflorescences to exogenous BA, we analyzed 27 transcripts in our dataset that were homologous to *Arabidopsis* genes involved in the cytokinin signal transduction pathway (Additional file 3). The expressions of *ARR3* and *ARR8* were upregulated, and *IPT5* was downregulated during the experiment, implying that exogenous cytokinin promoted increased ARR expression, which contributed to signal perception and transmission, and repressed the activation of IPTs that catalyze the first key reaction in endogenous cytokinin biosynthesis in the cytokinin signaling pathway. These findings were consistent with the results of other studies involving cytokinin treatment [46-48]. However, some genes that play important roles in cytokinin signaling, such as *ARABIDOPSIS HISTIDINE KINASE 3* (*AHK3*), *ARABIDOPSIS HISTIDINE-CONTAINING PHOSPHOTRANSFER 2* (*AHP2*), *ARR1*, *HOMEOBOX PROTEIN KNOTTED-1 LIKE 3* (*KNAT3*), *STM*, and *IPT9*, showed only small changes in their transcript levels, indicating that only small changes in the expressions of these genes are required to carry out their functions, rather than them being insensitive to exogenous BA. Many transcription factors are expressed at low levels; therefore, it was difficult to accurately assess their changes using the microarray method [46,49]. In addition, *ACETYL-COA CARBOXYLASE 1* (*ACC1*), *ARABIDOPSIS HEMOGLOBIN 2* (*AHB2*), *ARABIDOPSIS THALIANA HOMEOBOX PROTEIN 2* (*ATHB2*), and *QUASIMODO2* (*QUA2*) were also induced by the application of BA; however, their roles in cytokinin signaling are unclear.

### Cross-talk of cytokinin with other phytohormones

Eight major types of phytohormones coordinate plant growth and development by modulating various cellular processes in response to intrinsic and environmental cues: abscisic acid (ABA), auxins, brassinosteroids (BRs), cytokinins (CKs), ethylene, gibberellins (GAs), jasmonic acid (JA) and salicylic acid (SA) [47]. To understand the roles of other phytohormones in inflorescence buds in response to BA treatment, we identified homologous genes involved in various hormonal regulation pathways in *Arabidopsis* by sequence comparison between our set and the Arabidopsis Hormone Database [48]. In addition to the 27 cytokinin-related genes, 104 transcripts were identified in our dataset, including 23 abscisic acid-related genes, 32 auxin-related genes, 11 brassinosteroid-related genes, 11 ethylene-related genes, nine gibberellin-related genes, 11 jasmonic acid-related genes, and seven salicylic acid-related genes (Additional file 3).

In the ABA signaling pathways, *ABSCISIC ACID RECEPTOR* (*ABAR*) and *PYRABACTIN RESISTANCE-LIKE 4* (*PYL4*) encode two ABA receptors that are involved in perceiving ABA signals [50,51]. In this study, the transcript levels of *PYL4* and *ABAR* were downregulated by 3.5-fold at the 3-h time point and by 20.6-fold at the 12-h time point, respectively. *ABSCISIC ACID 1* (*ABA1*), which encodes a zeaxanthin epoxidase, which catalyzes the conversion of zeaxanthin to antheraxanthin and violaxanthin to generate the epoxycarotenoid precursor in the ABA biosynthetic pathway [52], was downregulated at the 12-h time point. Also, *1-DEOXY-D-XYLULOSE-5-PHOSPHATE SYNTHASE* (*DXS*), which encodes a key enzyme catalyzing a limiting step in the biosynthesis of plastidic isoprenoids (the carotenoid precursors for ABA biosynthesis) [53], was downregulated 4-fold at the 3-h time point. Moreover, *9-CIS-EPOXYCAROTENOID DIOXYGENASE 3* (*NCED3*), which encodes a key enzyme in the ABA biosynthesis pathway [54], was downregulated 3.8-fold at the 12-h time point. These results showed that BA treatment inhibited ABA signaling by repressing the expression of genes involved in ABA biosynthesis and ABA perception. This indicated that endogenous cytokinins might play similar roles in inhibiting the effects of ABA and maintaining reproductive growth during flower bud development in *J. curcas*.

In auxin signaling pathways, *MASSUGU2* (*MSG2/IAA19*) encodes an auxin-regulated protein that regulates hypocotyl growth and the formation of lateral roots together with AUXIN RESPONSE FACTOR7 (ARF7) [55]; it showed 2-fold upregulation at the 12-h time point. *IAA CARBOXYL METHYLTRANSFERASE 1* (*IAMT1*), which methylates indole-3-acetic acid (IAA) to form methyl-IAA ester and overexpression of which causes dramatic hyponastic leaf phenotypes [56], was downregulated 2.1-fold at the 12-h time point. *SUPERROOT 1* (*SUR1*) encodes a C-S lyase, which is involved in indolic glucosinolate biosynthesis and whose mutant showed a high-auxin phenotype related to the accumulation of indole-3-acetaldoxime promoted IAA biosynthesis [57], was downregulated 4.3-fold at the 12-h time point. *TRANSPARENT TESTA 4* (*TT4*) encodes a chalcone synthase that is the first enzyme in flavonoid biosynthesis and is an auxin transport inhibitor [58]; it was downregulated 2.8-fold at the 12-h time point. Jones *et al.* found that the application or ectopic biosynthesis of cytokinin rapidly induced an auxin increase in young shoot and root tissues and proposed that cytokinin promoted auxin synthesis by controlling the transcription of certain auxin biosynthesis genes [59]. However, our results suggested that cytokinin treatment caused the increase in auxin levels via suppression of the negative regulators of auxin biosynthesis pathways. In addition, the expression of *MYB77*, a positive regulator of auxin signaling transduction [60], was downregulated at the 48-h time point, indicating that auxin signaling was inhibited then, contradicting our conclusion that cytokinins promoted an increase in auxin. Therefore, we hypothesized that the downregulation of *MYB77* might help the accumulation of auxin in inflorescence buds by inhibiting auxin transport, indicating that the roles of MYB77 might be different in *Arabidopsis* and *J. curcas*.

*A* UDP-glycosyltransferase *gene (UGT73C5)* encode an enzyme that catalyzes the glucosylation of BRs, causing their inactivation [61]; it was downregulated 5-fold at the 12-h time point. *CONSTITUTIVE PHOTOMORPHOGENESIS AND DWARFISM* (*CPD/DWF3*), which encodes a cytochrome P450 steroid side-chain hydroxylase that plays an essential role in BR biosynthesis [62], was upregulated 3-fold at the 3-h time point. These results indicated that cytokinin caused an increase of BRs by repressing BR glucosylation and promoting BR biosynthesis, which repressed the expression of *FLC* to promote flowering in *Arabidopsis* [63].

In the ethylene signaling pathway, *MULTIPROTEIN BRIDGING FACTOR 1C* (*MBF1c*) encodes a coactivator that enhances plant tolerance to biotic and abiotic stresses, and is involved in both salicylic acid and ethylene signaling pathways [64]; it was upregulated by more than 4-fold during the time course of the experiment, especially at the 24-h time point (37.4-fold). *HIGH INDOLIC GLUCOSINOLATE 1* (*HIG1/MYB51*), which encodes a key transcription factor in indolic glucosinolate biosynthesis and responds to ethylene stimuli [65,66], was upregulated at the 3-h time point, and downregulated at the 12-h time point. These results indicated that *MBF1c* and *HIG1/MYB51* are candidate cross-talk genes in cytokinin and ethylene signaling pathways.

*GIBBERELLIN 20-OXIDASE 1* (*GA20ox1*) was upregulated between 3 h and 48 h, and *GIBBERELLIN-INSENSITIVE DWARF 1b* and *1c* (*GID1b* and *1c*) were upregulated at the 48-h time point; however, *GIBBERELLIN 2-OXIDASE 1* (*GA2ox1*) was downregulated between 12 h and 48 h. *GA20ox1* encodes an enzyme that, along with gibberellin 3β-hydroxylase, catalyzes the formation of active gibberellins, and *GID1s* encode gibberellin receptors that are positive regulators in the gibberellin signaling pathway [67,68]. GA2ox1 functions in a major catabolic pathway that negatively regulates gibberellin signaling [69]. The results showed that cytokinin promoted gibberellin production by increasing the transcription of *GID1s* and *GA20ox1* and decreasing that of *GA2ox* in inflorescence buds.

ABNORMAL INFLORESCENCE MERISTEM 1 (AIM1), ALLENE OXIDE SYNTHASE (AOS/CYP74A), OPR3 (OXOPHYTODIENOATE-REDUCTASE 3), and SUPPRESSOR OF SA-INSENSITIVITY 2 (SSI2/FAB2) are positive regulators of jasmonic acid signaling in *Arabidopsis* [70-74]: they were all upregulated after BA treatment. An AIM1 mutant had an abnormal floral meristem phenotype with severe sterility, and a knockout mutant of the *AOS* gene was male sterile in *Arabidopsis* [70,71], indicating that cytokinins promote an increase in jasmonic acid during *J. curcas* flower development. However, *JMT*, which encodes a jasmonic acid carboxyl methyltransferase that catalyzes the methylation of jasmonic acid [74], and is a positive regulator, was downregulated between 3 h and 48 h, implying that methyl jasmonate might play different roles in the JA signaling pathway in *J. curcas* and *Arabidopsis*. Moreover, BENZOIC ACID CARBOXYL METHYLTRANSFERASE 1 (BSMT1) catalyzes the methylation of salicylate and benzoate in the salicylic acid signaling pathway in response to biotic and abiotic stresses [75]. *BSMT1* was upregulated at the 3-h time point, indicating that methyl salicylate is involved in the response to BA treatment.

Based on these results, we concluded that exogenous BA influences the effects of major phytohormones by modulating the expression levels of genes involved in various metabolic pathways in *J. curcas*. These phytohormones may jointly regulate the development of *J. curcas* flowers after BA treatment, although their exact roles in this process remain to be defined. Elucidating the mechanisms of cross-talk among multiple signaling pathways is also essential.

## Conclusions

The application of BA could significantly increase flower number and seed yield in *J. curcas*. To elucidate the mechanism underlying this response, we performed a transcriptional analysis of *J. curcas* inflorescence buds after BA treatment. 5,555 differentially expressed transcripts were identified, which could be grouped into 12 clusters representing distinct regulatory patterns and belonged to 32 gene ontology categories. Based on our analysis of genes involved in flowering and phytohormone signaling pathways, we hypothesized that BA application increased flower number by activating the transcription of genes that initiate flowering and repressing that of genes involved in the formation of floral organs. Moreover, exogenous cytokinin treatment could influence the production of major phytohormones by regulating the transcription of genes involved in their metabolic pathways. BA treatment repressed endogenous cytokinin biosynthesis and abscisic acid signaling and promoted auxin, brassinosteroid, gibberellin, jasmonic acid, and salicylic acid signaling, suggesting that these plant hormones might jointly regulate the development of *J. curcas* flowers. Our study provides a basis for understanding the molecular mechanisms underlying changes in flowering traits in response to cytokinin treatment in *J. curcas*, and provides useful information on the mechanisms of cross-talk among multiple hormone signaling pathways in woody plants.

## Materials and methods

### Plant materials and treatments

*Jatropha curcas* L. is a cultivated plant in Yunnan Province, China [76]. One-year-old *J. curcas* plants were grown in a field at a density of 2 × 2 m per plant at Xishuangbanna Tropical Botanical Garden of the Chinese Academy of Sciences, located at Menglun town in Mengla County, Yunnan Province, China (21°54' N, 101°46' E, 580 m asl). To select a suitable concentration of the synthetic cytokinin 6-benzylaminopurine (BA) for treating *J. curcas*, 180 inflorescence buds (about 0.5–1 cm in diameter) were selected and distributed into five treatment groups, each of which included 36 inflorescence buds. Working solutions of various concentrations of BA (0, 0.5, 1.0, 2.0, or 4.0 mM) were sprayed onto inflorescence buds with a hand sprayer, wetting the inflorescence buds to the point of run-off. Tween-20 (Polysorbate-20, Shanghai Sangon Biological Engineering Technology & Services Co., Ltd., Shanghai, China) was added to the BA working solutions at a final concentration of 0.05% (v/v) as a wetting agent. The total flower number, female flower number, ratio of female to male flowers, fruit number, fruiting rate, seed number, seed yield per inflorescence, and weight of 100 seeds and seed oil content were surveyed during the development period.

For the microarray analysis, inflorescence buds were treated with 1.0 mM BA solution containing 0.05% Tween-20. Inflorescence buds were collected at 0, 3, 12, 24, and 48 h after BA treatment. The collected samples of inflorescence buds were frozen immediately in liquid nitrogen and stored at -80°C until RNA extraction. Three biological replicates were performed for each time point. The experiments were carried out in May 2010.

### Collection of sequences and design of probes

41,735 genetic sequences were collected (Additional file 4). Among them, 30,184 expressed sequence tags (ESTs) were generated in our laboratory by sequencing cDNA libraries of *J. curcas* flower buds and embryos [77]. The other sequences were publicly available from NCBI (up to 2010). 8,157 unigenes were produced from 16,875 ESTs derived from different *J. curcas* tissues [78,79] and 3,394 unigenes from 5,619 ESTs from castor bean flowers. For the *J. curcas* transcript data set, 41,651 sequence-specific probes of 60-bp oligonucleotides were designed using the Agilent eArray software (Additional file 5).

### RNA extraction, hybridization, microarray data acquisition, normalization and analysis

Total RNA was extracted from inflorescence buds using TRIzol (Invitrogen, Carlsbad, CA, USA). The RNA concentration was determined using a NanoDrop ND-1000 (Thermo Scientific, Waltham, MA, USA). RNA integrity was confirmed with an Agilent 2100 Bioanalyzer (Agilent, Santa Clara, CA, USA), and the total RNA was purified with a Qiagen RNeasy kit (Venlo, Netherlands). Two micrograms of RNA were reverse-transcribed to cDNA using a one-step method. The cDNA was transcribed into RNA by T7 RNA polymerase, modified by aa-UTP at 40°C, purified with a Qiagen RNeasy mini kit and quantified using the Bioanalyzer. Four micrograms of cRNA were labeled with Cy3 fluorescence dye at 25°C and purified with a Qiagen RNeasy mini kit. Eight hundred and seventy-five nanograms of Cy3 cRNA were fragmented and hybridized with 4 × 44 k arrays. After hybridization, the arrays were washed according to the manufacturer’s instructions and scanned twice using an Agilent scanner, with 10% and 100% photo multiplier tubes (PMT). Raw data from arrays were normalized by log_2_ transformation and analyzed using GeneSpring GX software (Agilent). Differentially expressed probes with fold-change thresholds ≥2 and corrected *P*-values ≤0.05 were selected. Hierarchical clustering analysis was performed using Cluster software, and the results viewed using the Java TreeView software [80,81].

### Assembly and annotation of sequences

Sequences corresponding to differentially expressed probes were assembled by CAP3 (Sequence Assembly Program) software [82]. The differentially expressed unigenes were annotated using Interproscan (version 4.8) [83], and the GO annotation results were plotted by WEGO (Web Gene Ontology Annotation Plot) [84].

### Validation of gene expression by qRT-PCR

qRT-PCR was performed on a LightCycler 480 II (Roche, Penzberg, Germany) using the SYBR green fluorescent label. The cDNA was synthesized from total RNA using a PrimeScript RT Reagent Kit (Takara, Otsu, Japan). The relative expression levels of genes were calculated by the 2^−ΔΔ^ CT method. All quantitative PCRs were repeated in 2–3 biological replications. The primers used for qRT-PCR are listed in Additional file 6.

### Availability of supporting data

Oligonucleotide microarray data have been deposited into the Gene Expression Omnibus (GEO) Database under accession number GSE54366 at http://www.ncbi.nlm.nih.gov/geo/query/acc.cgi?acc=GSE54366. All additional data files supporting the results of this article are available in the LabArchives repository and are accessible via http://dx.doi.org/10.6070/H4P848WW.

## Additional files

Additional file 1:
**10,569 probes representing 5,555 differentially expressed transcripts identified using microarray analysis.**
Additional file 2:
**Hierarchical clustering of differentially expressed transcripts.**
Additional file 3:
**Expression analysis of transcripts related to major phytohormones in** ***J. curcas.***
Additional file 4:
**41,735 sequences collected for the transcriptional analysis using microarray technology.**
Additional file 5:
**41,651 probes designed using Agilent eArray software.**
Additional file 6:
**Oligonucleotides primers used for qRT-PCR validation of selected transcripts from microarrays and for expressional analysis of** ***LFY,*** ***SOC1*** **and** ***TFL1b.***
